# Flexible Bronchoscopy in the Intensive Care Unit: Controversies, Clinical Applications, and the Expanding Role of Intensivists

**DOI:** 10.3390/jcm15124568

**Published:** 2026-06-12

**Authors:** Thushira Weerawarna, Rajesh Mishra, Sumara Tantray, Manish Bharti, Atul Mehta, Semra Bilaceroglu, Gaurav Mishra, Ahsina Jahan, Antonio Esquinas

**Affiliations:** 1 Siloah St. Trudpert Klinikum, 75179 Pforzheim, Germany; thushiraw@gmx.de; 2 Shaibya Comprehensive Care Clinic, Ahmedabad 380054, India; 3 Apollo Hospitals, New Delhi 110076, India; sumaira.tantray@gmail.com; 4 Metro Group of Hospital, Noida 110092, India; dr_manish_mamc@yahoo.co.in; 5 Cleveland Clinic Foundation, Cleveland, OH 44195, USA; mehtaa1@ccf.org; 6 Izmir Dr. Suat Seren Training and Research Hospital for Thoracic Medicine and Surgery, University of Health Sciences, 35100 Izmir, Turkey; s.bilaceroglu@gmail.com; 7 Smt. NHL Medical College, Ahmedabed 380006, India; gauravmishra1432002@gmail.com; 8 MH Samorita Hospital and Medical College, Dhaka 1208, Bangladesh; lopaafnan@gmail.com; 9 Intensive Care Unit, Hospital Morales Meseguer, International NIV School, 30008 Murcia, Spain; antmesquinas@gmail.com

**Keywords:** flexible bronchoscopy, intensive care unit, acute respiratory failure, bronchoalveolar lavage

## Abstract

**Background:** Flexible bronchoscopy (FB) has long been integral to pulmonology, but its bedside role in the intensive care unit (ICU) is expanding. Despite a lack of high-level evidence, FB remains a pivotal tool for airway visualization, sampling, and selected interventions in critically ill patients. **Objective:** This meta-narrative review critically appraises the clinical use, evolving indications, safety profile, and emerging controversies of FB in ICU settings, particularly regarding the role of non-pulmonologist intensivists. Methods: A structured literature search was conducted using PubMed, Scopus, and Google Scholar for studies published in the past 15 years. Emphasis was placed on observational studies, meta-analyses, and guidelines relevant to FB in ICU patients. Key controversies were grouped under thematic questions based on clinical relevance. **Results:** A total of 84 articles were retrieved, of which 47 met the predefined inclusion criteria. Seven key thematic domains were synthesized regarding the use of flexible bronchoscopy (FB) in the intensive care unit (ICU) setting. FB performed by trained intensivists was found to be safe and diagnostically effective across a range of ICU populations, including elderly and non-intubated patients. Although procedure-related hypoxemia was reported, it was largely manageable with appropriate precautions. FB demonstrated critical utility in the management of acute respiratory failure (ARF), acute respiratory distress syndrome (ARDS), and sepsis, particularly through bronchoalveolar lavage (BAL), airway secretion clearance, and, selectively, bronchoscopic lung biopsy. The adoption of disposable bronchoscopes may reduce infection risk and economic burden. Furthermore, the integration of advanced techniques such as endobronchial ultrasound (EBUS) and transbronchial cryobiopsy is emerging, although application in the critical care environment remains cautious and selective. **Conclusions:** With structured training and careful patient selection, FB is an adaptable and often underutilized tool in ICU medicine. Multidisciplinary competency development and institutional protocols can enhance its safe integration.

## 1. Introduction

Flexible bronchoscopy (FB) has been an essential diagnostic and therapeutic modality in pulmonary medicine since its introduction in the 1960s. Its early adoption into critical care followed shortly thereafter, especially for secretion management and airway inspection in intubated patients. Despite this long-standing role, the bulk of evidence supporting FB use in the intensive care unit (ICU) derives from observational studies, case series, and expert consensus rather than randomized trials [1,2].

A previous study noted that the core indications for FB in the ICU—diagnosis of pneumonia, airway clearance, and support for intubation—have changed little over decades [3]. However, recent developments such as rapid molecular diagnostics, single-use bronchoscopes, and minimally invasive interventional techniques are now expanding the potential applications of FB in critical care.

It is also crucial to distinguish between basic bronchoscopy (airway visualization, bronchoalveolar lavage [BAL], secretion clearance) and advanced interventional bronchoscopy, which includes cryobiopsy, endobronchial ultrasound (EBUS), and bronchial stenting. While pulmonologists traditionally perform these advanced procedures, the boundaries are increasingly overlapping as trained intensivists gain procedural competence.

This review explores the utility, safety, controversies, and training considerations for FB in ICU patients, with a specific focus on whether trained non-pulmonologists should perform FB, and under what conditions FB offers diagnostic or therapeutic superiority in critical illness.

The rationale for this review extends beyond cataloguing established bronchoscopic indications. The modern ICU landscape is characterised by evolving procedural boundaries between pulmonologists and intensivists, an increasing demand for bedside interventions in resource-limited and off-hours settings, and ongoing institutional debates regarding competency-based training frameworks. Furthermore, the emergence of single-use bronchoscopes, rapid molecular diagnostics on bronchoalveolar lavage (BAL) fluid, endobronchial cryobiopsy, and endobronchial ultrasound (EBUS) is fundamentally reshaping what is technically feasible at the bedside. These developments render a contemporary re-examination of the role of flexible bronchoscopy in the ICU not merely relevant but necessary. Rather than summarising established applications, this review critically examines the controversies, contradictions, and evolving responsibilities surrounding FB in the critical care environment—including questions of procedural ownership, credentialing, and the appropriate delineation of tasks between generalist intensivists and interventional subspecialists.

## 2. Materials and Methods

This review was conducted in accordance with a meta-narrative review framework, guided by the principles of the RAMESES publication standards for meta-narrative reviews [4]. A structured literature search was performed across multiple electronic databases, including PubMed, Google Scholar, and Scopus, covering the period from January 2008 to March 2024. Relevant studies were identified using predefined search terms such as “flexible bronchoscopy,” “intensive care unit,” “bronchoalveolar lavage,” “bronchoscopy in ARDS,” “FB complications ICU,” “disposable bronchoscope,” “cryobiopsy,” “EBUS ICU,” and “bronchoscopic lung biopsy.” The inclusion criteria encompassed peer-reviewed original research articles, meta-analyses, systematic reviews, clinical guidelines, and expert commentaries focusing on the use of flexible bronchoscopy in adult ICU settings, while paediatric studies, animal studies, and editorials lacking supporting data were excluded. Older studies exceeding 10 years were selectively included if they were foundational or if recent high-quality evidence was limited. All retrieved articles were screened based on clinical relevance, methodological rigor, and their contribution to ongoing debates in the field. The identified literature was then thematically organized around key clinical considerations commonly encountered in ICU practice, including operator expertise, age-related tolerance, safety, cost-effectiveness, and its applications in acute respiratory distress syndrome or acute respiratory failure.

A RAMESES-guided meta-narrative framework was chosen given the heterogeneous, multidisciplinary evidence base spanning critical care, pulmonology, interventional pulmonology, thoracic anaesthesia, and airway management—with predominantly observational literature and substantive contradictions around procedural ownership, safety, and training. The review evolved iteratively, with recurring controversies around credentialing, workforce implications, and patient selection prompting targeted supplementary searches. Pre-2008 foundational studies were selectively included to honour the historicity principle, particularly for technologies such as laser bronchoscopy (1981), APC (1994), endobronchial valves (2003), and linear EBUS (2004). Perspectives from multiple specialties were actively incorporated to reflect the contested nature of the themes examined.

### Results of Literature Search

A total of 84 articles were identified through searches of PubMed, Scopus, and Google Scholar using the predefined search strategy. Following deduplication, 71 unique records were screened by title and abstract, of which 47 met the predefined inclusion criteria and were included in the final synthesis. The excluded articles were either paediatric studies, animal studies, editorials without supporting data, or studies not directly addressing flexible bronchoscopy in the adult ICU setting.

**Figure 1:**
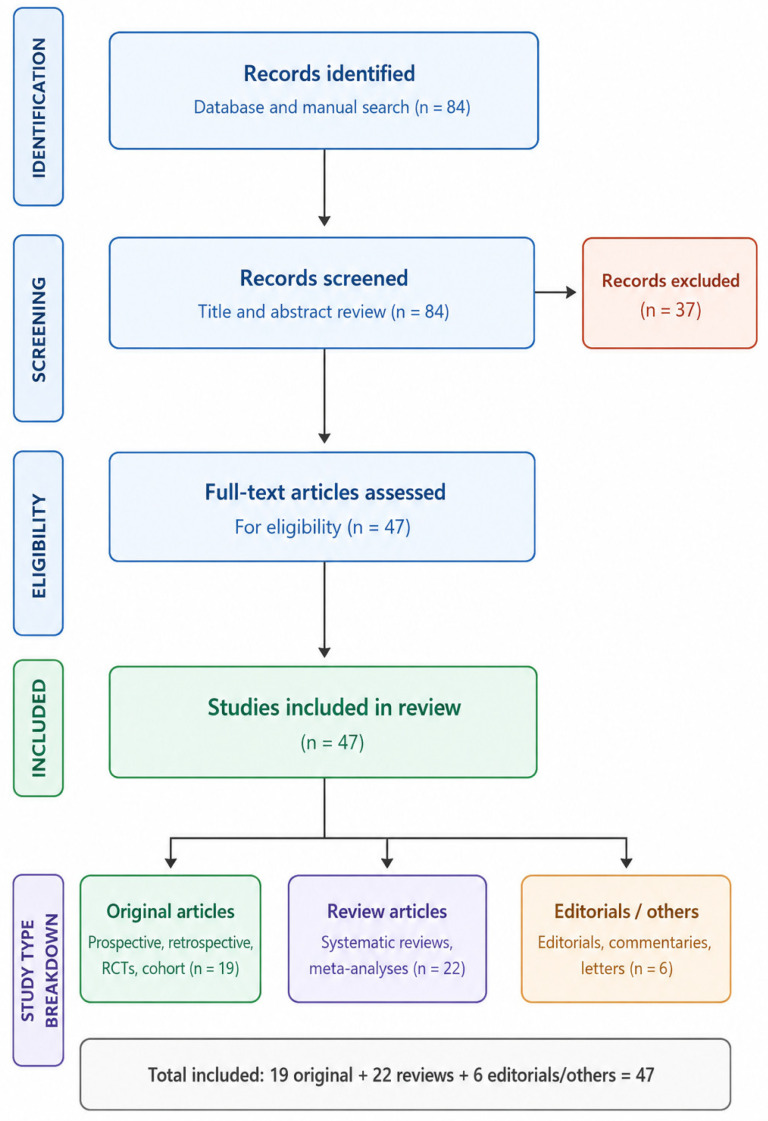
PRISMA Flow Diagram.

The included literature comprised 18 observational cohort studies (prospective and retrospective), 6 meta-analyses or systematic reviews, 9 clinical practice guidelines or expert consensus documents, 8 case series or retrospective ICU-based studies, and 6 meta-narrative or thematic review articles. Where available, multi-centre prospective studies were prioritised. Foundational studies predating the 2008 search cutoff were selectively retained where they represented historically significant contributions or where more recent high-quality evidence was absent. A PRISMA flow diagram is provided in Figure 1.

The review evolved iteratively across three phases. Initially, focus centered on safety and diagnostic utility in intubated patients. As screening progressed, unresolved controversies around procedural ownership and intensivist credentialing emerged, prompting targeted supplementary searches. Our initial assumption that elderly and non-intubated patients were poor candidates for FB was revised after multiple prospective studies demonstrated acceptable safety with appropriate precautions. Finally, the growing literature on single-use bronchoscopes, rapid molecular diagnostics, and advanced techniques (EBUS, cryobiopsy) emerged as an unanticipated but substantive thematic domain, leading to expansion of the advanced techniques section.

## 3. Themes

### 3.1. Can Non-Pulmonologists Perform Flexible Bronchoscopy Safely in ICU Patients?

The question of whether non-pulmonologist intensivists can safely perform flexible bronchoscopy (FB) in critically ill patients is central to the ongoing debate on procedural autonomy in the ICU. This discussion is rooted in concerns about patient safety, procedural efficacy, and the timeliness of intervention.

#### 3.1.1. Evidence Supporting Non-Pulmonologists Performing FB

Critically ill patients often deteriorate rapidly and require urgent diagnostics or airway interventions. Delaying bronchoscopy for pulmonologist availability may adversely impact outcomes, especially in resource-limited or off-hour settings [1]. Studies have demonstrated that intensivists trained in FB can perform the procedure safely, achieving comparable diagnostic yield and complication rates to pulmonologists [2,3].

In many European training programmes, bronchoscopy is now an expected competency for critical care fellows [4]. A study by Fuehner et al. showed that establishing a 24-h emergency bronchoscopy service—largely staffed by non-pulmonologists—was associated with improved patient care and reduced delays [5].

The American College of Chest Physicians (ACCP) guidelines propose a minimum of 50 supervised procedures for establishing bronchoscopy competence, a number now integrated into several critical care fellowship curricula [6]. Intensivists commonly use FB for BAL, secretion clearance, and airway inspection—especially when managing difficult airways, post-intubation bleeding, or unexplained hypoxemia.

#### 3.1.2. Evidence Highlighting Limitations

However, concerns remain regarding procedural tolerance, particularly in non-intubated ICU patients. A multicenter prospective study by Kamel et al. found that operator inexperience, defined as fewer than 50 bronchoscopies or fewer than 10 years of ICU experience, was independently associated with poorer tolerance and higher complication rates in non-intubated patients (odds ratio 3.57; 95% CI, 1.04–12.35) [7].

Additionally, non-pulmonologists may lack advanced interventional skills needed during complex procedures such as managing massive hemoptysis or performing cryobiopsy, potentially limiting their scope of practice. This has led to suggestions that planned FB in non-intubated patients or high-risk cases should be reserved for experienced bronchoscopists, regardless of specialty, to optimise safety.

#### 3.1.3. Balancing Perspectives

The practical reality is that critical care units often operate with limited pulmonology coverage. Structured training programmes, credentialing, and simulation-based bronchoscopy education have been shown to improve procedural safety and operator confidence across specialities [8].

Thus, a balanced view emerges: intensivists can safely perform FB when appropriately trained and credentialed, particularly in emergent or basic diagnostic procedures. However, complex therapeutic interventions or non-intubated cases may benefit from referral to pulmonologists or interventional bronchoscopists when feasible.

### 3.2. Is Flexible Bronchoscopy Well-Tolerated in Elderly ICU Patients?

With the global population aging and elderly individuals accounting for an increasing proportion of ICU admissions, understanding the safety and utility of flexible bronchoscopy (FB) in this group is crucial. The elderly are more vulnerable to respiratory illnesses and often require prompt airway assessment and intervention due to age-related impairments in cough reflex, mucociliary clearance, and immune function [9,10].

#### 3.2.1. Risks and Physiological Considerations in the Elderly

Advanced age is associated with reduced pulmonary reserve, increased risk of aspiration, and higher susceptibility to complications from invasive procedures. Neurological comorbidities such as stroke, delirium, and Parkinson’s disease, common in the elderly, further predispose patients to respiratory deterioration and extubation failure [11,12].

While concerns persist regarding procedural tolerance, especially with sedation and hypoxemia, multiple studies have shown that FB can be safely performed in elderly patients when proper precautions are followed.

#### 3.2.2. Evidence Supporting Safety

A prospective study comparing patients aged ≥85 years and 65–79 years found no significant difference in complication rates, suggesting age alone is not a predictor of adverse outcomes [13]. Similar findings have been reported in both retrospective [14,15] and prospective studies [16], indicating a low incidence of severe complications—including bleeding, pneumothorax, and post-procedural fever.

Moreover, FB in elderly ICU patients has shown diagnostic and therapeutic benefits, particularly in cases of lobar collapse due to mucus plugging. Bronchoscopic suctioning under direct vision, coupled with mucolytics or lavage, improves oxygenation and often alleviates respiratory distress [17].

#### 3.2.3. Clarifying Contradictions

While data on frailty-specific outcomes is limited, age should not be viewed as a contraindication to bronchoscopy. Rather, the decision to perform flexible bronchoscopy (FB) in elderly patients should be individualized, taking into account the patient’s functional and physiological status, the severity of the underlying respiratory illness, and a careful assessment of the anticipated procedural benefits relative to potential risks. This nuanced approach resolves the apparent contradiction noted in earlier drafts: although elderly patients may have increased physiological vulnerability, existing evidence supports the safe use of FB with appropriate patient selection and monitoring.

### 3.3. What Is the Safety Profile of Flexible Bronchoscopy in Critically Ill Patients?

Flexible bronchoscopy (FB) is generally considered safe in the intensive care setting when performed for appropriate indications and by adequately trained operators. Nonetheless, concerns persist regarding the risk of complications, especially in severely hypoxemic or hemodynamically unstable patients.

#### 3.3.1. Reported Complication Rates

Complications associated with FB in ICU patients include hypoxemia, arrhythmias, hypotension, bleeding, and—in rare cases—cardiac arrest or pneumothorax. However, the overall incidence of major complications remains low, with mortality rates below 0.01% and serious adverse events ranging from 0.08% to 2% [18,19]. A large retrospective study of 332 bronchoscopies in a respiratory ICU reported hypoxemia in 3.6%, hypotension in 1.5%, bradycardia in 1.2%, and bleeding in 1.2% of cases [20].

In non-intubated patients, the use of non-invasive ventilation (NIV) or high-flow nasal cannula (HFNC) during FB has been shown to reduce the risk of desaturation, making the procedure feasible even in patients with moderate to severe hypoxemia [21,22,23,24].

#### 3.3.2. Infection Risk and the Role of Disposable Scopes

Recent attention has turned to the potential for FB to act as a vector for nosocomial infections, especially when reusable bronchoscopes are inadequately disinfected. Damaged channels or improper reprocessing may facilitate the transmission of multi-drug-resistant organisms (MDROs) in the ICU [25].

To mitigate this risk, single-use disposable bronchoscopes have been introduced. These eliminate the need for sterilization and may reduce cross-contamination, though evidence is mixed on cost-effectiveness and image quality [26,27]. Their use has become increasingly common, particularly in high-risk or immunocompromised ICU populations.

#### 3.3.3. Monitoring and Safety Protocols

Pre-procedural planning, optimization of oxygenation, correction of coagulopathy, and continuous monitoring (including heart rate, SpO_2_, blood pressure, and end-tidal CO_2_) significantly reduce the risk of adverse events during FB. Protocol-driven practice enhances safety, especially in mechanically ventilated or hemodynamically fragile patients [28].

Ultimately, while complications can occur, most are mild, reversible, and manageable with appropriate precautions. The safety profile of FB supports its use in the ICU, especially when weighed against its diagnostic and therapeutic benefits.

### 3.4. Is Bronchoscopy Redundant When Imaging Studies Are Available?

While modern imaging modalities such as computed tomography (CT), magnetic resonance imaging (MRI), and lung ultrasound provide invaluable information about thoracic structures, they do not render flexible bronchoscopy (FB) obsolete in ICU care. Instead, these tools serve complementary roles.

Imaging allows non-invasive visualization of lung parenchyma, pleura, and large airways. However, only FB enables direct airway inspection, targeted sampling through bronchoalveolar lavage (BAL) or brushings, and therapeutic interventions, such as mucus plug removal or hemostasis in active hemoptysis [29,30].

In unstable ICU patients, the need for intrahospital transport to radiology for CT imaging carries its own risks, including ventilator disconnections, desaturation, and hemodynamic compromise [31,32]. By contrast, FB can be safely performed at the bedside, making it especially valuable when urgent diagnostics or airway interventions are required.

Moreover, FB allows access to dynamic airway conditions (e.g., tracheomalacia, excessive dynamic airway collapse), which are not easily appreciated on static imaging [33]. It also plays a crucial role when imaging findings are inconclusive or discordant with clinical signs.

Thus, while imaging and FB often overlap in indications, bronchoscopy does not replace imaging, nor is it replaced by it. Rather, it remains essential for direct airway visualization, tissue sampling, and timely bedside intervention.

### 3.5. Is the Cost/Benefit Ratio of Bronchoscopy Favourable in ICU?

Flexible bronchoscopy (FB) entails direct procedural costs, particularly when reusable scopes are employed—necessitating cleaning, disinfection, and maintenance. A 2022 meta-analysis estimated the cost of a reusable bronchoscopy procedure at around $266 USD [26]. In contrast, disposable bronchoscopes offer logistical advantages and lower the risk of cross-contamination, though they may not always be cost-effective in low-volume centers [25,27].

Despite these upfront costs, FB has the potential to reduce overall ICU expenditures by facilitating timely diagnosis of infections through bronchoalveolar lavage (BAL), enabling earlier and more targeted interventions. It also contributes to a reduction in ventilator days by aiding in the clearance of mucus plugs and supporting clinical decision-making regarding extubation readiness. Furthermore, FB can help avoid the unnecessary use of broad-spectrum antibiotics by allowing pathogen-specific therapy, thereby improving antimicrobial stewardship [34,35].

For instance, bronchoscopy performed early in patients with aspiration pneumonia or suspected ventilator-associated pneumonia (VAP) has been associated with shorter ICU stays and improved antimicrobial targeting [36,37]. However, some studies show neutral effects on mortality, indicating that patient selection is key [38].

Cost-effectiveness also depends on institutional experience and operator proficiency. The integration of rapid molecular diagnostics (e.g., multiplex PCR panels on BAL samples) may improve diagnostic yield but must be supported by appropriate antibiotic stewardship to impact clinical outcomes meaningfully [39].

In summary, while FB introduces a measurable cost, its diagnostic and therapeutic utility in select ICU patients justifies its use. Strategic integration with infection control protocols and targeted use of single-use scopes can enhance its value proposition.

### 3.6. Should Flexible Bronchoscopy Be Used in Acute Respiratory Failure (ARF) and ARDS?

#### 3.6.1. Acute Respiratory Failure (ARF)

Acute respiratory failure (ARF) in ICU patients is often caused by pneumonia, aspiration, pulmonary haemorrhage, drug toxicity, malignancy, or exacerbations of chronic lung disease. When the aetiology is unclear, bronchoscopy—especially with bronchoalveolar lavage (BAL)—can aid diagnosis and guide targeted therapy.

The clinical utility of flexible bronchoscopy (FB) is especially evident in specific subgroups, including immunocompromised individuals such as those with hematologic malignancies or transplant recipients, patients presenting with persistent pulmonary infiltrates despite negative non-invasive evaluations, and those with suspected invasive fungal, mycobacterial, or atypical infections [40,41,42]. The use of rapid diagnostic tests (RDTs)—such as multiplex PCR assays on BAL fluid—has enhanced the diagnostic speed and accuracy of FB in ARF. These tests can detect viral, bacterial, and fungal pathogens within hours. However, when applied to non-invasive samples (e.g., sputum or nasopharyngeal swabs), RDTs may yield similar results, challenging the routine need for FB in all ARF cases [39,43].

A randomized study conducted in cancer patients with acute respiratory failure (ARF) demonstrated that non-invasive rapid diagnostic tests (RDTs) were non-inferior to bronchoscopy with bronchoalveolar lavage (FB-BAL) in detecting pathogens [44]. Accordingly, the use of flexible bronchoscopy (FB) should be more selectively considered and reserved for situations where non-invasive RDTs yield negative or inconclusive results, in patients who continue to show clinical deterioration despite appropriate empirical therapy, or when there is a strong suspicion of dual pathology or mixed infections.

#### 3.6.2. Acute Respiratory Distress Syndrome (ARDS)

ARDS presents a more complex challenge. The pathophysiology involves diffuse alveolar damage and capillary leak, with increased risk of hypoxemia during FB. Nonetheless, FB can still play a diagnostic or therapeutic role in selected cases, especially early in the disease course.

Its use is especially relevant in cases where aspiration pneumonia is suspected as an underlying cause of ARDS, when atelectasis or mucus plugging contributes to worsening hypoxia, or when there is diagnostic uncertainty and alternative conditions that mimic ARDS—such as eosinophilic pneumonia or alveolar haemorrhage—need to be excluded [45].

**Figure 2:**
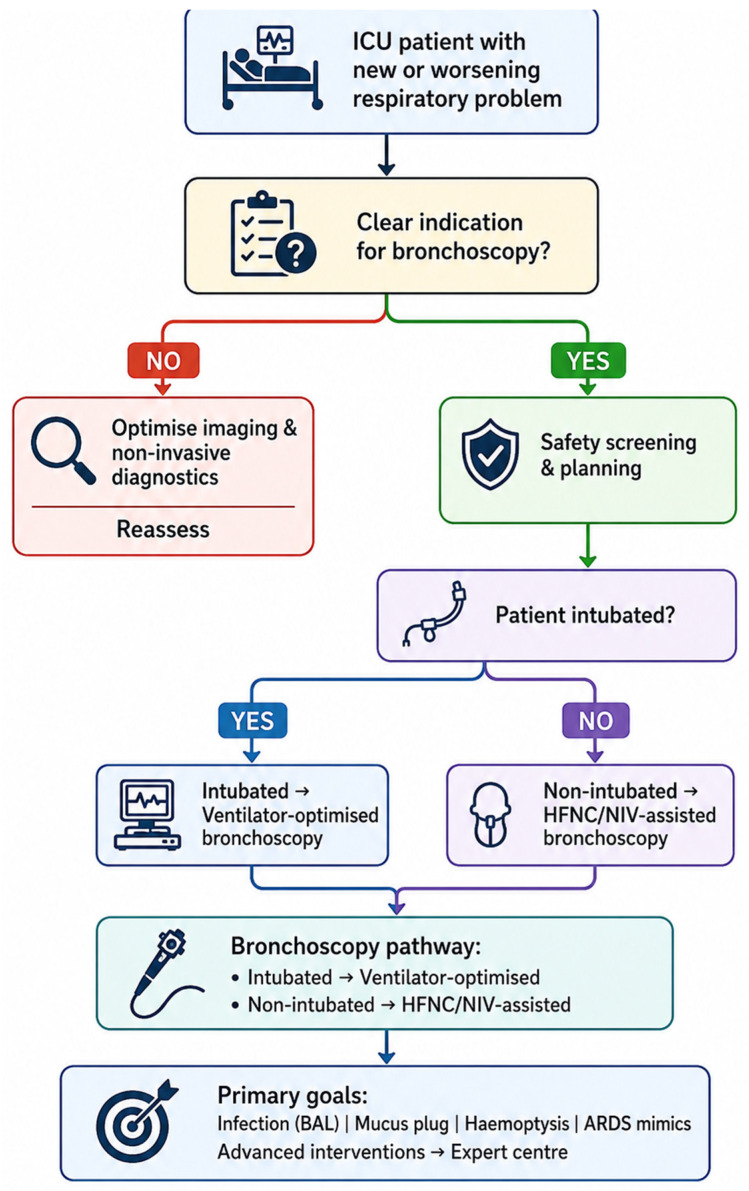
Point of Care Flexible Bronchoscopy Decision Algorithm (**Abbreviations:** ICU = Intensive Care Unit; HFNC = High-Flow Nasal Cannula; NIV = Non-Invasive Ventilation; ARDS = Acute Respiratory Distress Syndrome; BAL = Bronchoalveolar Lavage).

In mechanically ventilated patients, FB and BAL were not associated with increased complications, including in studies involving prone positioning. However, BAL in severe ARDS must be cautiously performed, given the risk of decruitment and worsening gas exchange. Figure 2 can help in deciding for doing point of care bronchoscopy in acute respiratory failure.

In this context, FB should generally be avoided when non-invasive diagnostic methods are sufficient to identify the causative pathogens, when the patient’s oxygenation status is marginal and unlikely to tolerate procedural desaturation, or when operator expertise is limited and the clinical indication for the procedure is not strong.

### 3.7. What Is the Role of Advanced Bronchoscopic Techniques in ICU Practice?

While flexible bronchoscopy (FB) in the ICU traditionally focused on airway inspection and bronchoalveolar lavage (BAL), recent years have witnessed the cautious introduction of advanced bronchoscopic techniques for diagnostic and therapeutic applications in critically ill patients. These methods, more common in interventional pulmonology, are increasingly relevant for selected ICU scenarios.

#### 3.7.1. Endobronchial Ultrasound (EBUS)

EBUS allows real-time sampling of mediastinal and hilar lymph nodes via transbronchial needle aspiration (EBUS-TBNA). In ICU patients with suspected malignancy, sarcoidosis, lymphoma, or tuberculosis, EBUS has shown high diagnostic yield with a favourable safety profile [46].

Although the setup may be more complex in critically ill patients, bedside EBUS is feasible in intubated or sedated individuals. In one series, EBUS modified clinical management in nearly 60% of cases, supporting its selective use when standard imaging is inconclusive and tissue is needed urgently.

#### 3.7.2. Cryobiopsy

Transbronchial cryobiopsy provides larger, more intact tissue samples than conventional forceps biopsy, thereby improving histopathologic diagnostic yield. It has proven useful in undifferentiated interstitial lung disease, diffuse alveolar damage, and suspected organizing pneumonia.

In a small study of intubated ICU patients, cryobiopsy showed an acceptable safety profile, with complications mainly limited to manageable bleeding and a low incidence of pneumothorax [47]. However, this technique demands specific training, bronchial blockers, and rapid availability of resuscitative support.

In massive hemoptysis, bronchoscopic balloon occlusion using a Fogarty catheter or endobronchial blocker can isolate the bleeding airway and protect the contralateral lung, serving as a critical temporizing measure pending bronchial artery embolization or surgery [29,30]. Balloon dilation (bronchoplasty) is applicable for post-intubation tracheal or subglottic stenosis, restoring airway patency at the bedside without surgical intervention.

Flexible bronchoscopy also serves as an essential rescue tool in difficult airway management. When conventional laryngoscopy fails, bronchoscope-guided intubation remains the gold-standard technique. ICU teams should maintain bronchoscopy within their difficult airway algorithm, with trained operators available around the clock and simulation-based training integrated into fellowship curricula [6,8].

#### 3.7.3. Navigational and Robotic Bronchoscopy

Technologies such as electromagnetic navigation and robotic bronchoscopy enable high-precision access to peripheral lung lesions. While these are primarily used in outpatient settings for diagnosing solitary pulmonary nodules, their future application in the ICU may be considered for critically ill cancer patients, where a delay in tissue diagnosis impacts prognosis. Despite this potential, several practical limitations restrict their routine use in critical care settings, including limited equipment availability, procedural and setup complexity, and challenges related to sedation and ventilation in hemodynamically unstable patients. Consequently, these advanced bronchoscopic techniques are not yet widely adopted in routine ICU practice.

#### 3.7.4. Therapeutic Interventions: Stents and Endobronchial Valves

In selected critically ill patients, flexible bronchoscopy (FB)-guided therapeutic interventions can provide significant, and at times life-saving, clinical benefit. These include airway stent placement, which can restore and maintain airway patency in conditions such as central airway obstruction due to tumors or tracheomalacia, thereby improving ventilation. Additionally, endobronchial valves have emerged as a minimally invasive option for the management of persistent air leaks, including bronchopleural fistula and post-lobectomy air leaks, even in mechanically ventilated patients. The successful implementation of these interventions necessitates a multidisciplinary approach involving intensivists, pulmonologists, and interventional specialists, along with appropriate imaging guidance such as fluoroscopy or computed tomography (CT) correlation. Careful post-procedural monitoring is essential to ensure optimal outcomes and to promptly identify potential complications.

#### 3.7.5. Limitations: Advanced Bronchoscopic Interventions

Despite promising data, the application of advanced bronchoscopic interventions in the intensive care unit (ICU) remains limited and is often considered off-label. This is primarily due to the lack of robust controlled trials specifically conducted in critically ill populations, which restricts the generalizability of existing evidence. Additionally, the availability of skilled operators and specialized equipment is often limited, particularly in resource-constrained settings. Procedural challenges further arise from concerns related to prolonged procedure duration, the need for deeper levels of sedation, and the potential impact on ventilation in hemodynamically unstable patients. Consequently, these factors collectively limit the routine adoption of advanced bronchoscopic techniques in critical care practice.

#### 3.7.6. Limitations of Meta-Narrative Review:

As a meta-narrative review, this article is inherently limited by non-systematic selection of studies, which may introduce selection bias despite an effort to include relevant and recent publications. While the thematic approach aligns with real-world clinical questions, it may underrepresent contradictory evidence or emerging data not captured through conventional indexing.

The majority of cited studies on flexible bronchoscopy (FB) in the ICU are observational, retrospective, or single-center case series. Few randomized controlled trials exist, and generalizability is further limited by heterogeneous patient populations, variable operator experience, and institution-specific protocols.

Further statistical uncertainty arises from inconsistencies in the definition and reporting of complications, such as hypoxemia and bleeding, as well as variability in patient selection, including differences between mechanically ventilated and non-intubated individuals. In addition, procedural heterogeneity—such as the use or absence of fluoroscopic guidance and differences in biopsy techniques—adds to the complexity of interpreting outcomes. Many studies also lack standardized outcome reporting, and meta-analyses frequently combine heterogeneous endpoints, which may lead to either overestimation or underestimation of the true safety and effectiveness of FB in critical care settings.

Lastly, while this review includes some references over 10 years old, these were retained only when more recent evidence was lacking or when they represented foundational studies still widely cited in contemporary clinical practice.

## 4. Conclusions

Flexible bronchoscopy (FB) remains a valuable tool in intensive care medicine, offering both diagnostic and therapeutic benefits at the bedside. Despite its long-standing presence in the ICU, the practice continues to evolve—driven by technology, training innovations, and the need for rapid, tailored interventions in critically ill patients.

The accumulated evidence supports the safe and effective use of FB by trained non-pulmonologist intensivists, particularly for airway inspection, bronchoalveolar lavage (BAL), secretion clearance, and urgent diagnostic procedures. While operator inexperience can compromise tolerance and safety in non-intubated patients, structured training and credentialing can mitigate this risk. Age alone should not be considered a contraindication to FB.

Complications are generally mild and manageable, with hypoxemia being the most common. When performed under appropriate conditions—with patient selection, monitoring, and supportive respiratory strategies—FB is well tolerated. The use of disposable bronchoscopes may reduce cross-contamination risk and streamline logistics, particularly in immunocompromised patients.

FB complements rather than replaces imaging. It also enables advanced interventions such as cryobiopsy, EBUS, and even therapeutic procedures in select ICU patients. While these techniques remain specialized, their role is expanding as ICU teams acquire broader procedural capabilities. Ultimately, FB in the ICU should be approached with clarity of indication, procedural planning, and team-based expertise. As training standards and evidence evolve, FB is poised to become not just a pulmonologist’s tool, but a core component of critical care practice—empowering intensivists to act swiftly and precisely in complex, time-sensitive situations. Procedural ownership disputes can be addressed through tiered competency frameworks—basic skills (BAL, airway inspection, secretion clearance) as core ICU competencies, while advanced techniques (cryobiopsy, EBUS-TBNA, stenting) remain with interventional specialists. Procedural risk reflects patient physiological instability rather than bronchoscopy itself, making careful patient selection preferable to blanket restriction. Standardised competency-based training and minimum supervised procedure thresholds would reduce the inter-institutional variability in outcomes currently observed.

### Future Directions

Future directions involve miniaturized scopes, improved monitoring tools, and integration of advanced FB techniques into structured ICU airway programs with standardized protocols and training pathways.

Future research should aim for multicentre prospective trials, with protocolized FB use, clear safety endpoints, and comparison against emerging non-invasive diagnostic strategies.

## Data Availability

No new datasets were generated for this meta-narrative review. All data analyzed were obtained from previously published studies cited within the manuscript.

## Abbreviations

The following abbreviations are used in this manuscript:

ACCP	American College of Chest Physicians
ARDS	Acute Respiratory Distress Syndrome
ARF	Acute Respiratory Failure
BAL	Bronchoalveolar Lavage
COVID-19	Coronavirus Disease 2019
CT	Computed Tomography
EBUS	Endobronchial Ultrasound
EBUS-TBNA	Endobronchial Ultrasound–Transbronchial Needle Aspiration
FB	Flexible Bronchoscopy
HFNC	High-Flow Nasal Cannula
ICU	Intensive Care Unit
MDROs	Multidrug-Resistant Organisms
MRI	Magnetic Resonance Imaging
NIV	Non-Invasive Ventilation
RDTs	Rapid Diagnostic Tests
VAP	Ventilator-Associated Pneumonia
